# The Influence of Metakaolin and Polypropylene Fiber Concrete on Mechanics Properties and Microstructure Combined Action under Multi-Salt Soaking and Freeze–Thaw

**DOI:** 10.3390/ma16165525

**Published:** 2023-08-08

**Authors:** Yongwei Gao, Borui Zhou, Xianhua Yao, Junfeng Guan, Xiaoyu Han

**Affiliations:** 1 School of Mechanical Engineering, North China University of Water Resources and Electric Power, Zhengzhou 450011, China; gaoyongwei@ncwu.edu.cn; 2 School of Civil Engineering and Communication, North China University of Water Resources and Electric Power, Zhengzhou 450011, China; mzhouborui@126.com (B.Z.); junfengguan@ncwu.edu.cn (J.G.);; 3 State Key Laboratory of Eco-Hydraulics in Northwest Arid Region, Xi’an University of Technology, Xi’an 710048, China

**Keywords:** multi-salt soaking and freeze–thaw, potable water and freeze–thaw, metakaolin, polypropylene fiber, microstructure

## Abstract

The wide distribution of alpine saline areas in China is faced with two major problems, which are salt intrusion and freeze–thaw. In total, 216 specimens were prepared with 6 kinds of concrete mix proportions in this paper. The effects of the single and compound incorporation of metakaolin (MK) and polypropylene fiber (PPF) of different amounts on the mechanical properties and microstructure properties of concrete were investigated under the dual action of multi-salt (NaCl, MgCl_2_, Na_2_SO_4_, and NaHCO_3_) soaking and freeze–thaw. Potable water and freeze–thaw concrete were adopted as the control group. Changes in the appearance morphology, mass loss, relative dynamic elastic modulus, and compressive strength of the concrete were tested, and the microstructure of the concrete was analyzed by scanning electron microscopy (SEM). The results showed that an admixture of both MK and PPF in the potable water and freeze–thaw cycle test can improve the mechanical properties and frost resistance of concrete. The admixture of PPF can effectively improve the mechanical properties and frost resistance of concrete. However, the admixture of MK failed to improve the mechanical properties and frost resistance of concrete during multi-salt soaking and freeze–thaw. The frost resistance of concrete under multi-salt soaking and freeze–thaw was optimally improved with 10% MK and 0.6 kg/m^3^ PPF. Its microstructure shows that PPF can effectively inhibit crack propagation and reduce the deterioration of concrete under multi-salt soaking and freeze–thaw.

## 1. Introduction

Saline soil is widely distributed in the northwest and northeast of China. The damage caused by chloride, sulfate, magnesium salt, and carbonate to concrete is becoming more and more serious. In addition, freeze–thaw cycles in high/cold environments aggravate the corrosion damage of concrete. In order to solve the problem of the corrosion damage of concrete in alpine saline soil areas, adding mineral admixtures and fiber materials to improve the durability of concrete is the most commonly used low-cost technical measure [1,2].

Mineral admixtures represented by silica fume have been widely used in concrete [3,4,5], but the construction costs are too high, which is not conducive to popularization and application. Many studies have found that the performance of MK is similar to that of silica fume [6,7]. Therefore, scholars have carried out a series of studies on the incorporation of MK into concrete. Zheng et al. [5,8,9] believe that MK has a certain activity and can produce a good composite effect with other active mineral admixtures. The incorporation of 10–15% MK can improve the interfacial structure of lightweight aggregate concrete, accelerate the secondary hydration reaction of cement, and can thus greatly improve the frost resistance of lightweight aggregate concrete. Asteris et al. [10] developed a computational model for estimating the compressive strength of concrete with MK using the artificial neural network (ANN) technique. The model was able to reproduce to some extent the nonlinear effect of mix composition on concrete strength to obtain the optimum percentage of MK replacement. For early strength, the optimum replacement level is between 10% and 15%. Hassan et al. [11] believe that the addition of 5~10% silicon powder and MK can improve the durability of pervious concrete. Lachemi et al. [12,13] found that with the increase in the MK dosage, the skin spalling of the specimens was significantly reduced and the frost resistance was gradually enhanced, and the optimum dosage of MK was 20%. At the same dosage, the frost resistance of MK is better than that of silica fume.

To overcome the expansion of internal cracks in concrete, domestic and foreign scholars have incorporated fibers into concrete for modification. The addition of fibers is an effective way to improve the early crack resistance of concrete [14,15,16]. In addition, the distribution of fibers can well alleviate the expansion of cracks in concrete [17]. The research results of Teng et al. [18] show that 0.75~1.5% PPF can effectively reduce the loss of relative dynamic elastic modulus and effectively improve flexural strength, but the effect on compressive strength is not significant. Kurpińska et al. [19,20] concluded that natural and composite fibers have a 24–27% positive effect on the compressive strength of cementitious composites. Cheng et al. [21] believe that the compressive strength of concrete will be significantly improved when the PPF content is in the range of 0.6 kg/m^3^~1.5 kg/m^3^, and with the increase in PPF content, the strength damage gradually decreased. Li et al. [22] believe that the addition of PPF can reduce the porosity of large pores (d ≥ 500 μm) and improve the pore structure of concrete. Zhou et al. [23] explored the effects of different types and dosages of fibers on the frost resistance of concrete. They believe that the addition of fibers can improve the frost resistance of concrete, and the frost resistance of concrete increases with the increase in fiber content in a certain range. Cavdar [24] conducted freeze–thaw cycle tests on five different types of fiber cement mortars: PPF, carbon fiber, aramid fiber, glass fiber, and polyvinyl alcohol fiber. He believed that the fiber improved the flexural strength of the mortar and reduced the compressive strength, dynamic elastic modulus, and mass loss. Yan et al. [25] added an air-entraining agent to PPF concrete, performed a rapid freeze–thaw cycle test on the PPF concrete in potable water, and believed that freeze–thaw damage increases with the increase in freeze–thaw time. Under the same freeze–thaw cycles, the addition of an air-entraining agent played a positive role in improving the frost resistance of concrete.

In summary, current research on the freeze–thaw test for fibers and MK incorporated into concrete is mostly focused on the freeze–thaw test under potable water and is less involved in the freeze–thaw test under multi-salt soaking. Concrete under salt erosion and freeze–thaw will face the erosion of corrosive ions and freeze–thaw damage at the same time. The effect has a more complex failure mechanism, which accelerates the process of the deterioration and attenuation of concrete performance. Therefore, based on the engineering practice of the Delingha–Xiangride expressway in Qinghai Province (in China), composite multi-salt soaking was configured according to the concentration of the solution in the field. Considering the incorporation of MK and PPF into concrete, the effects of MK content (0, 10%, and 15%) and PPF content (0, 0.6 kg/m^3^, 0.9 kg/m^3^, and 1.5 kg/m^3^) on the compressive strength, mass loss rate, relative dynamic elastic modulus of concrete and its influence on the internal microstructure of concrete were designed under multi-salt soaking with freeze–thaw and potable water with freeze–thaw. The failure mechanism and microstructure mechanism of concrete under multi-salt soaking with freeze–thaw conditions were analyzed, which provides a reference for the engineering design of high-cold saline–alkali corrosion areas.

## 2. Raw Materials and Test Methods

### 2.1. Raw Material

Cement: PO 42.5 ordinary Portland cement; its 3 days compressive strength is 30.7 MPa, and 28 days compressive strength is 59.2 MPa. The specific chemical composition is shown in Table 1.

**Figure 1:**
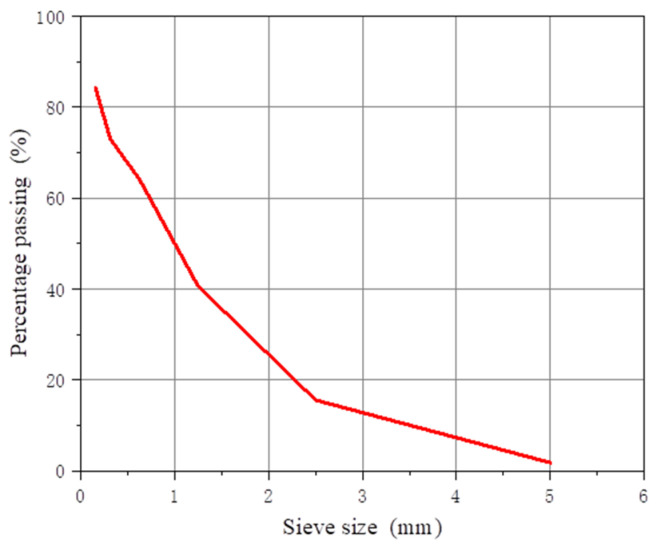
The grading curve of the fine aggregates.

Coarse aggregate: gravel with 5~10 mm continuous gradation; its bulk density is 1533 kg/m^3^, its particle density is 2686 kg/m^3^, and it has a rough surface and hard texture. Fine aggregate: medium sand; its fineness modulus is 2.74, its bulk density is 1550 kg/m^3^, its particle density is 2614 kg/m^3^, and its particle gradation is good, as shown in Figure 1. Water: potable water. 

Water reducer: carboxylic acid superplasticizer was used, the water reduction rate was 28%, and the other indicators meet the requirements of the specification. Because the incorporation of MK and PPF will affect the working performance of concrete, the amount of water-reducing agent is changed according to the amount of MK and PPF to ensure the normal working performance of the concrete; air-entraining agent: the dosage was 0.007% of the amount of cementitious material.

**Figure 2:**
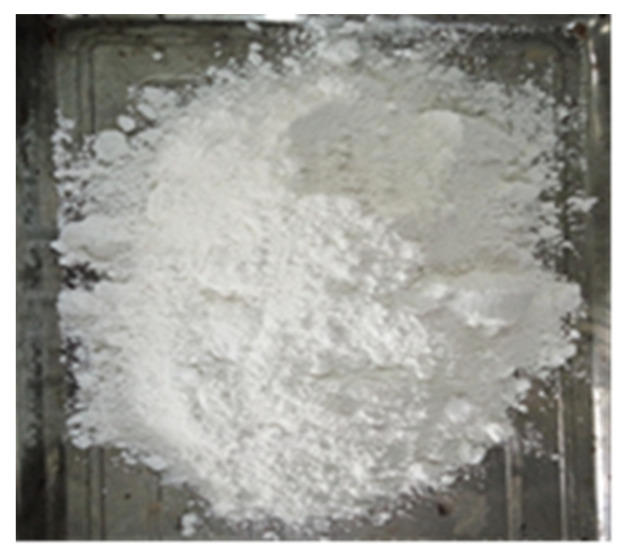
Metakaolin.

**Figure 3:**
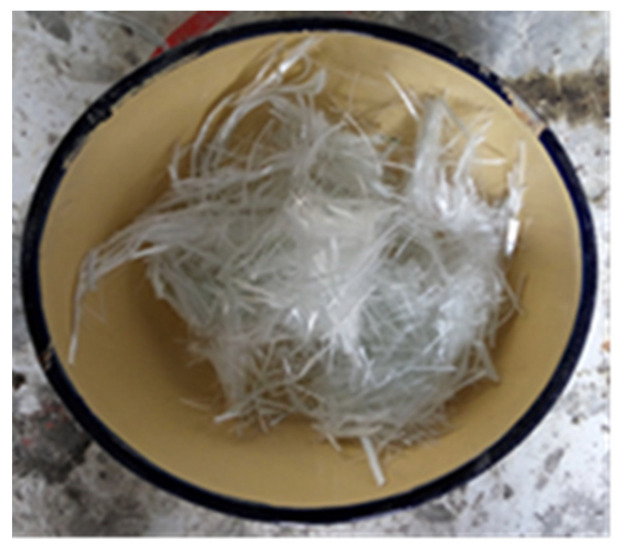
Polypropylene fiber.

MK is a white powder with a fineness of 4665 m^2^·kg^−1^ (Figure 2), and the specific chemical composition is shown in Table 1. PPF is made of tensile-resistant staple fiber (Figure 3) and is 19 mm in length and 30 μm in diameter. 

### 2.2. Preparation of Multi-Salt Soaking 

Four kinds of salts (NaCl, MgCl_2_, Na_2_SO_4_, and NaHCO_3_) were used to the prepare multi-salt solution based on the ion concentration in the construction site of De Xiang Expressway in Qinghai Province (Table 2). 

### 2.3. Mix Proportion and Test Method

The concrete mix is shown in Table 3. A total of 216 concrete specimens with 6 kinds of concrete mix ratios were used, with a size of 40 mm × 40 mm × 160 mm. Mixing was carried out by hand. The cement and coarse and fine aggregates were mixed and stirred for 1 min, and then, the well-mixed water, water-reducing agent, and air-entraining agent mixed solution was added to the mix three times for stirring. Finally, it was put into the test mold for shaping. After standard curing for 24 days, multi-salt soaking with freeze–thaw and potable water with freeze–thaw were carried out respectively. 

Multi-salt soaking and freeze–thaw test: After standard curing for 24 days, the concrete specimens were placed in multi-salt soaking, and the liquid level was 2 cm higher than the surface of the specimens; they were soaked for 4 days. Then, according to the “Hydraulic Concrete Test Procedure” (SL/T352-2020 [26]), the concrete specimens were moved into the rapid freeze–thaw test machine for the freeze–thaw cycle test.

**Figure 4:**
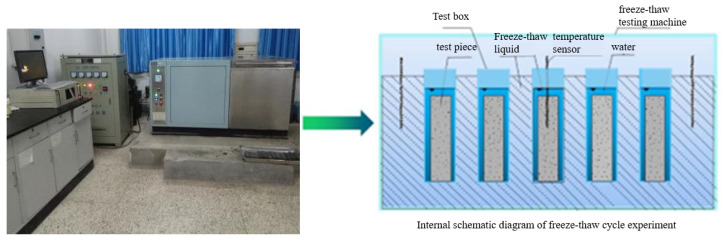
Freeze–thaw testing machine.

And then the concrete specimens were moved into a rapid freeze–thaw testing machine (Figure 4) for the freeze–thaw cycle test. Reference was made to the “Hydraulic Concrete Test Procedure” (SL/T352-2020). Each freeze–thaw cycle was 4 h. The center temperature of the specimen at the end of freezing and thawing was controlled at (−18 ± 2) °C and (5 ± 2) °C, respectively. The concrete mass loss, relative dynamic elastic modulus change, and compressive strength change were measured after every 25 freeze–thaw cycles test. The specific calculations are shown in Formulas (1)–(3).

(1) $$V=\frac{T}{L}$$

(2) $$E=\frac{\rho V_{}^{2}(1+\mu )(1-2\mu )}{1-\mu }$$

(3) $$Ed=\frac{E_{n}}{E_{0}}\times 100\%$$

In the formula, *V* is the longitudinal wave velocity of the ultrasonic wave propagating in the concrete, m/s; *T* is the ultrasonic propagation time and the unit is s; *L* is the longitudinal length of concrete, m; *E* is the dynamic elastic modulus of concrete in GPa; *μ* is Poisson’s ratio, and *μ* is generally 0.15 when the material is concrete; *ρ* is the density of concrete in kg/m^3^; *E_d_* represents the relative dynamic elastic modulus of concrete in%; *E_n_* represents the dynamic elastic modulus of the specimen after the freeze–thaw cycles, GPa; and *E*_0_ represents the dynamic elastic modulus of the specimen without freeze–thaw, GPa.

The potable water and freeze–thaw test: The control group of multi-salt soaking with the freeze–thaw test and the soaking solution is potable water. The other test methods are the same as the multi-salt soaking with the freeze–thaw test method.

## 3. Results Analysis and Discussion

### 3.1. Appearance of Specimen

(1) Effect of PPF content

**Figure 5:**
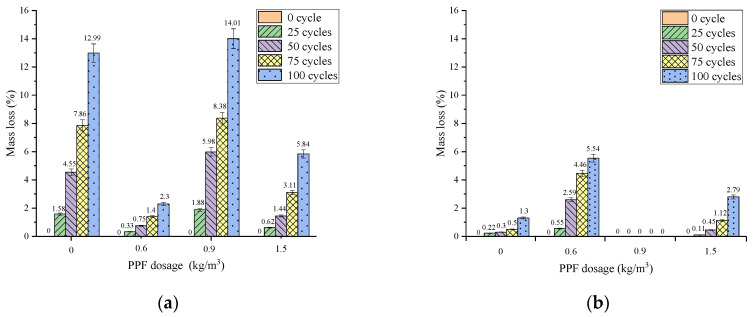
Relationship between mass loss and PPF dosage of concrete under different freeze–thaw cycles. (**a**) Multi-salt soaking and freeze–thaw and (**b**) potable water and freeze–thaw.

**Figure 6:**
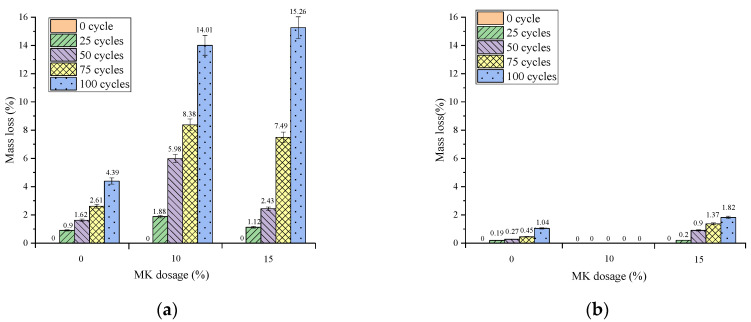
Relationship between mass loss and the MK dosage of concrete under different freeze–thaw cycles. (**a**) Multi-salt soaking and freeze–thaw and (**b**) potable water and freeze–thaw.

The concrete specimens were mixed with 10% MK (0 kg/m^3^ PPF, 0.6 kg/m^3^ PPF, 0.9 kg/m^3^ PPF, and 1.5 kg/m^3^ PPF) under the condition of multi-salt soaking and potable water. The pictures of the appearance after 0, 25, 50, 75, and 100 freeze–thaw cycles are shown in Figure 5 and Figure 6. The surface damage of the specimen is more serious under multi-salt soaking with freeze–thaw conditions. And the degree of surface damage increases with the number of freeze–thaw cycles. The proportion of the P-I, P-III and P-IV specimens changed 25 freeze–thaw cycles after surface mortar spalling due to pit erosion. The surface of the proportional P-II specimen is relatively flat and smooth (Table 4). The surface mortar of the specimens with the ratios P-I, P-III, and P-IV almost completely flaked off after 75 freeze–thaw cycles. Coarse aggregates were exposed and started to spall. The coarse aggregates in the specimen were exposed or even started to flake off and appeared to be missing corners. However, only a few cement pastes fell off and pitting corrosion appeared on the surface of the specimens with P-II. Therefore, the concrete with 0.6 kg/m^3^ PPF had better spalling resistance in the salt freezing test.

For the concrete specimens under potable water freezing and thawing changed with the increase in the number of freeze–thaw cycles, their skin gradually came off, revealing the internal aggregate (Table 5). And the surface damage to the specimen was lower than the specimen from multi-salt soaking with the freeze–thaw test. Among the four groups of specimens, the specimen with the P-IV ratio was the first to suffer obvious damage. The specimens with the ratio P-IV were partially peeled off and exposed the aggregate after 25 freeze–thaw cycles. And the surface of the specimen with the ratio P-III remained flat. After 75 freeze–thaw cycles, the skin of the specimens with the ratio P-IV all fell off and revealed the aggregate. For the surface of the specimen with the ratio P-III only part of the granular cement paste was dislodged. The specimens with the ratio P-I showed small defects with slight spalling on the surface at the end of 100 freeze–thaw cycles. And the specimen with ratio P-III was only slightly pockmarked. Therefore, the concrete mixed with 0.9 kg/m^3^ PPF had better anti-stripping performance in the potable water and freeze–thaw environment.

In summary, increasing the amount of fiber can effectively play a bridging effect on the concrete to block the shrinkage cracks. Fibers can assist cementitious materials to restrain the frost swelling stresses generated by the pores. But too many fibers entangled together will have an agglomeration effect. At this point, the fiber not only cannot play a bridging role but it also increases the internal pores of the concrete. However, too many fibers can have a negative effect on the freeze–thaw properties of the concrete. Excessive fiber entanglement will cause an agglomeration effect, which will not only fail to play a bridging role but will also increase the internal pores of the concrete.

(2) Effect of MK content

The concrete specimens were mixed with 0.9 kg/m^3^ PPF (0% MK, 10% MK, and 15% MK) under the condition of multi-salt soaking and potable water. The pictures of the appearance after the freeze–thaw cycles are shown in Table 6 and Table 7. After 25 times of multi-salt soaking with the freeze–thaw cycle test, the specimens were all pockmarked (Table 6); after 75 times of multi-salt soaking with freeze–thaw cycles, the surface of the specimens with M-I was relatively flat. The specimens with M-II and M-III were seriously damaged, with exposed aggregates and angular defects.

In the water freeze–thaw test, the surface of the specimen after the freeze–thaw cycles was smooth and flat (Table 7). After 50 freeze–thaw cycles, the surface cement paste of the specimens with M-I and M-III fell off, and the surface of the specimens with M-II remained smooth. Among them, the M-II specimen was only slightly pockmarked after 100 freeze–thaw cycles, while the cement paste of the proportioned M-I and M-III specimens fell off, and the aggregates were exposed. Therefore, under the condition of potable water and freeze–thaw, mixing with 10% MK can improve the spalling resistance of the concrete. The addition of MK does not improve the spalling resistance of the concrete under the condition of multi-salt soaking with freeze–thaw. This is mainly due to the fact that the large amount of silica contained in MK can undergo a volcanic ash effect to consume Ca(OH)_2_, but it reduces the alkalinity inside the concrete while generating hydrated calcium silicate gel. This is very detrimental to the concrete’s resistance to sulfate attack. Therefore, MK cannot improve the frost resistance of concrete in multi-salt solutions.

### 3.2. Quality

(1) Effect of PPF content

Concrete quality decreases after freeze–thaw cycles due to spalling damage. Quality loss rate can effectively reflect the appearance of concrete damage. The relationship between mass loss and the PPF dosage of concrete under different freeze–thaw cycles and different freeze–thaw conditions and PPF dosages are shown in Figure 6.

The mass loss rate of the concrete increased with the number of freeze–thaw cycles for each group under different conditions. And the mass loss rate of the concrete under multi-salt soaking under freeze–thaw conditions was significantly higher than that of potable and water freeze–thaw under the same number of freeze–thaw cycles. The image of the mass loss rate under multi-salt soaking with freeze–thaw conditions is shown in Figure 5a. The concrete with PPF dosing of 0.6 kg/m^3^ had the lowest mass loss rate. And the mass loss rate was much lower than the control group without PPF in all stages of the freeze–thaw cycles. When the number of cycles reached 100, the concrete mass loss rate of the control group was more than five times the mass loss rate of the concrete mixed with 0.6 kg/m^3^ PPF. This may be due to the better fluidity and more adequate hydration reaction of concrete mixed with 0.6 kg/m^3^ PPF. And this produces more calcium hydroxide, which can effectively resist the salt solution. When analyzing Figure 5b, the concrete with PPF dosing of 0.9 kg/m^3^ maintained the lowest mass loss rate at all stages of freeze–thaw cycles under the potable water and freeze–thaw conditions.

(2) Effect of MK content

The relationship between the mass loss rate and the MK dosing of concrete under different freeze–thaw conditions and different numbers of freeze–thaw cycles is shown in Figure 6. The mass loss rate of concrete increased with the number of freeze–thaw cycles for each group under different conditions. Compared with multi-salt soaking and freeze–thaw, the mass loss rate of concrete under potable and water freeze–thaw was relatively small. The mass loss rates of concrete mixed with MK were all higher than those without MK under multi-salt soaking with freeze–thaw conditions (Figure 6a). Therefore, under the condition of potable water freezing and thawing, with the increase in MK content, the mass loss rate does not change much (Figure 6b). Under multi-salt soaking with freeze–thaw conditions, the addition of MK to concrete will significantly increase its mass loss.

### 3.3. Relative Dynamic Elastic Modulus

(1) Effect of PPF content

**Figure 7:**
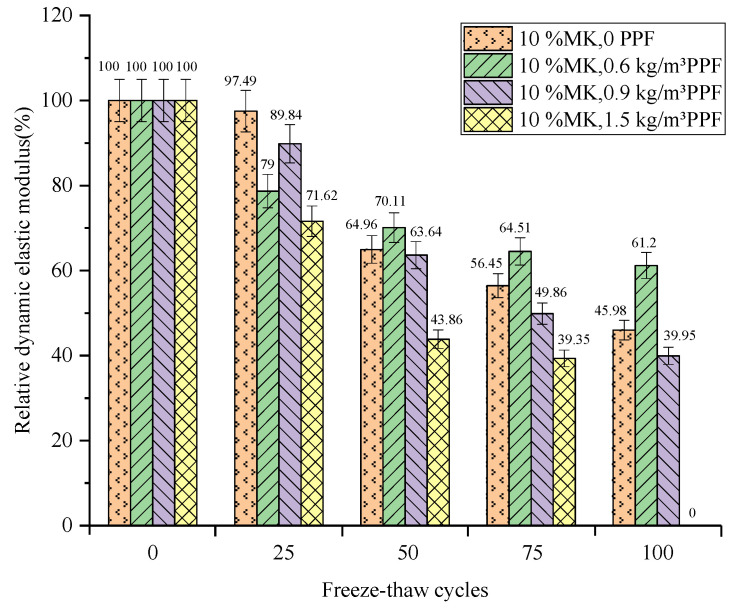
Relationship between the freeze–thaw cycles and the relative dynamic elastic modulus under multi-salt soaking and freeze–thaw.

**Figure 8:**
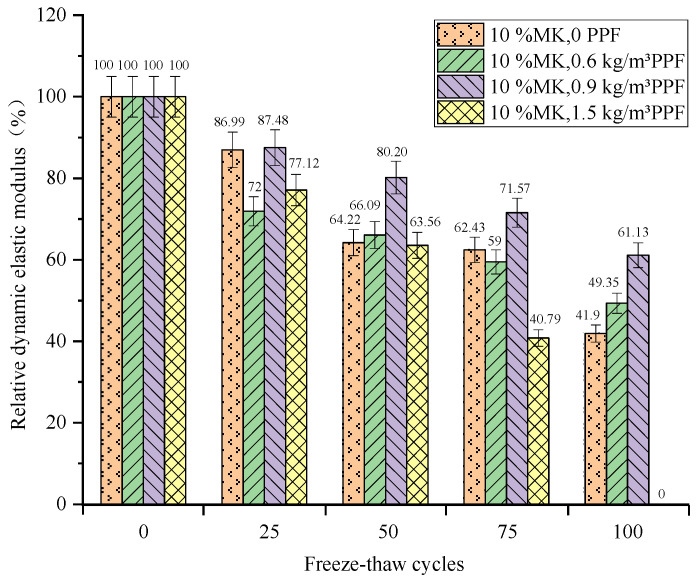
Relationship between the freeze–thaw cycles and the relative dynamic elastic modulus under potable water and freeze–thaw for PPF.

The relationship between the relative dynamic elastic modulus of concrete and the number of freeze–thaw cycles under different freeze–thaw conditions and PPF dosages is shown in Figure 7 and Figure 8. The relative dynamic modulus of elasticity of concrete decreases with the increasing number of freeze–thaw cycles under both conditions. And the relative dynamic elastic modulus loss values of concrete are similar under both conditions.

The relative dynamic elastic modulus loss under multi-salt soaking with freeze–thaw conditions decreases and then increases with the increase in PPF dosing. After 100 multi-salt soaking with freeze–thaw cycles (Figure 7), the relative dynamic elastic modulus loss of concrete with PPF content of 0.6 kg/m^3^ is the smallest (38.8%), which was lower than that of the control group (54.02%). The maximum loss of the relative dynamic modulus of elasticity (100%) was observed for concrete with PPF dosing of 1.5 kg/m^3^. Therefore, adding a certain amount of PPF to concrete under multi-salt soaking with freeze–thaw conditions can reduce the loss of relative dynamic elastic modulus. However, too much PPF will increase its freeze–thaw loss, and there is an optimal dosing amount (0.6 kg/m^3^) under multi-salt soaking with freeze–thaw conditions.

The relative dynamic elastic modulus loss of concrete decreases and then increases with the increase in PPF admixture in potable water and freeze–thaw conditions. Under the condition of potable and water freeze–thaw, with the increase of PPF content, the relative dynamic elastic modulus loss decreases first and then increases. After 100 freeze–thaw cycles (Figure 8), the relative elastic modulus loss of concrete with PPF content of 0.9 kg/m^3^ is the smallest (38.87%), which is lower than that of the control group (58.1%). The relative dynamic elastic modulus loss of concrete with PPF content of 1.5 kg/m^3^ is the largest (100%). Therefore, adding a certain amount of PPF to the concrete under the condition of potable and water freeze–thaw can reduce the loss of relative dynamic elastic modulus, but excessive dosage will increase its loss, and there is an optimal dosage (0.9 kg/m^3^) under the condition of potable water and freeze–thaw.

Comparing the two tests, the phenomenon of freeze–thaw damage under partial clear water solution appears more serious. This may be due to the fact that the addition of a small amount of salt solution will react with calcium hydroxide in concrete. The generated aqueous solution reacts with the unhydrated cement particles in the cement. Therefore, the content of calcium hydroxide in the concrete is reduced, making the interface transition zone harder and increasing the strength of the concrete.

(2) Effect of MK content

**Figure 9:**
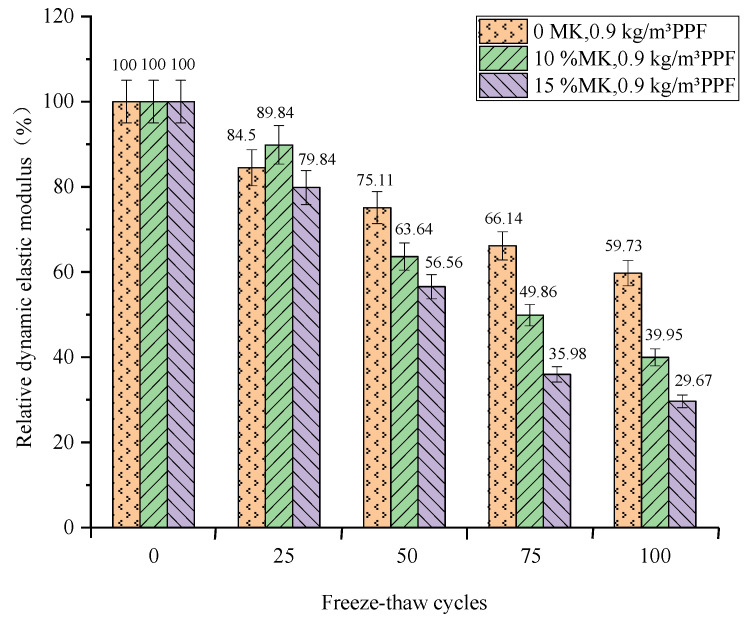
Relationship between freeze–thaw cycles and the relative dynamic elastic modulus under multi-salt soaking and freeze–thaw.

The relative dynamic elastic modulus loss increases with the increase of MK doping under multi-salt soaking and freeze–thaw conditions. After 100 freeze–thaw cycles (Figure 9), the control group had the smallest loss of relative dynamic modulus of elasticity (40.27%), which was lower than the experimental group mixed with MK. The maximum loss of relative dynamic modulus of elasticity was (70.33%) for concrete with 15% MK dosing. This may be due to the presence of Mg^2+^, SO_4_^2−^, and Cl^−^, which will cause erosion inside the concrete. Also, the concrete was mixed with 0.9 kg/m^3^ PPF, while the adhesion of the fiber to the cement paste was poor. This may provide a channel for the penetration of solutions and aggressive ions, making it easier to enter the concrete [27]. And the addition of MK under salt freezing conditions can effectively improve the resistance of concrete to Cl^−^ penetration. However, the corresponding mitigation effect of chloride salt on sulfate corrosion is also reduced. The chemical corrosion reactions of Mg^2+^ and SO_4_^2−^ on concrete promote each other, pushing its microcrack development and accelerating the freeze–thaw damage effect. 

**Figure 10:**
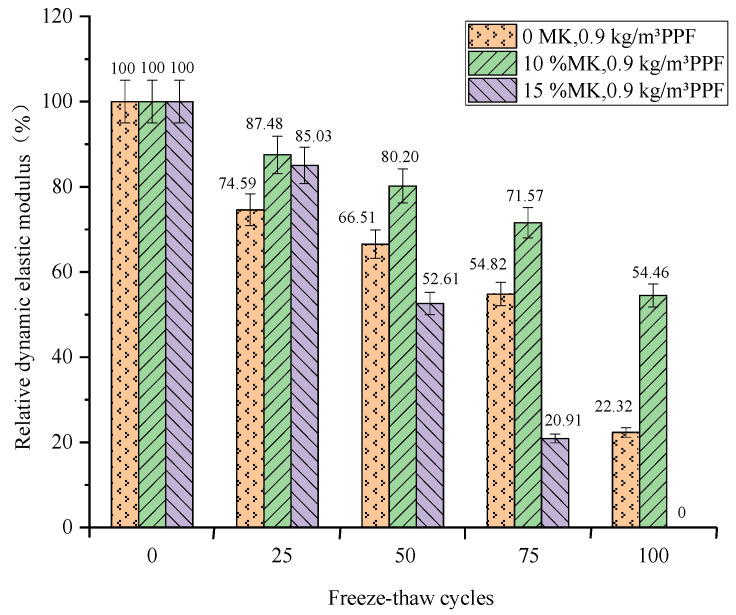
Relationship between the freeze–thaw cycles and the relative dynamic elastic modulus under potable water and freeze–thaw for MK.

Under the condition of potable and water freeze–thaw, with the increase in MK content, the relative dynamic elastic modulus loss decreased first and then increased. After 100 freeze–thaw cycles (Figure 10), the relative elastic modulus loss of concrete with 10% MK content is the smallest (45.54%), which is lower than that of the control group (77.68%). The relative dynamic elastic modulus loss of concrete with 15% MK content is the largest (100%). The addition of MK can improve the frost resistance of concrete under the condition of water freezing and thawing. This is because, in the water freezing and thawing cycle test, the high volcanic ash characteristics of MK make the internal structure of concrete dense and then improve the frost resistance of concrete. However, when the content exceeds 10%, the frost resistance of concrete does not improve. The chemical corrosion reactions of Mg^2+^ and SO_4_^2−^ on concrete promote each other, pushing its microcrack development and accelerating the freeze–thaw damage effect.

In summary, the addition of MK does not reduce the loss of relative dynamic modulus of elasticity of concrete under multi-salt soaking with freeze–thaw conditions. And the loss of relative dynamic elastic modulus increases with the increase in MK doping. The appropriate amount of MK can reduce the loss of relative dynamic modulus of elasticity of concrete under freeze–thaw conditions of potable water. However, too much MK will increase its loss, and there is optimal dosing (10%) under potable and water freeze–thaw conditions.

### 3.4. Compressive Strength

(1) Effect of PPF content

During the freeze–thaw cycle, concrete is subjected to freeze swelling, ice crystal pressure from NaCl and chemical corrosion. These factors can further develop their internal pre-existing cracks. With the increase in the number of freeze–thaw cycles, the cracks inside the specimen gradually increase and penetrate each other, ultimately reducing the load-bearing capacity of concrete [28].

**Figure 11:**
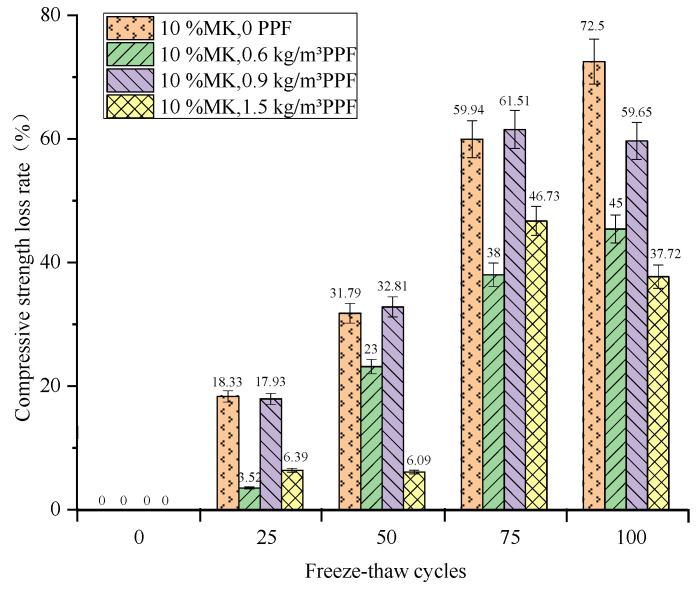
Relationship between freeze–thaw cycles and compressive strength loss rate under multi-salt soaking and freeze–thaw for PPF.

**Figure 12:**
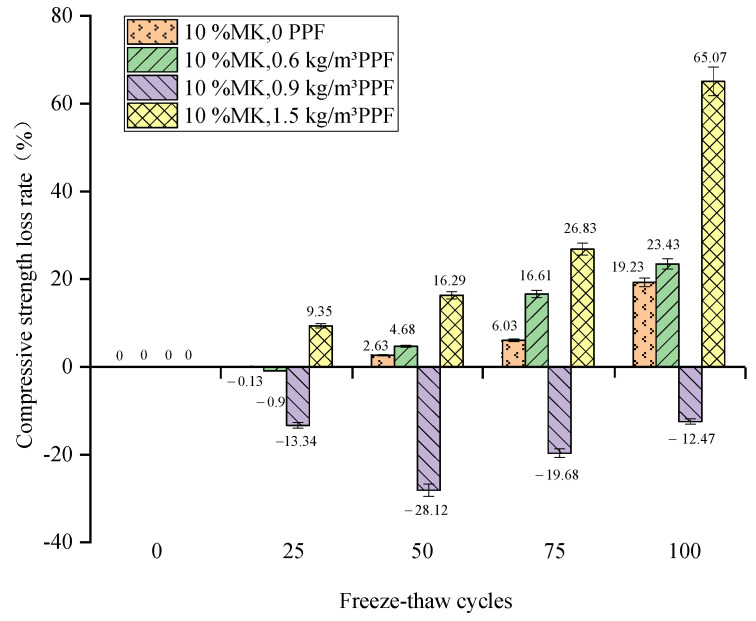
Relationship between the freeze–thaw cycles and the compressive strength loss rate under potable water and freeze–thaw (note: negative values indicate increments).

The relationship between the compressive strength loss rate and the number of freeze–thaw cycles of concrete under different freeze–thaw conditions and PPF dosages is shown in Figure 11 and Figure 12. From the overall trend, the compressive strength of concrete under multi-salt soaking with freeze–thaw and potable water freezing-thaw conditions decreases with the increase in the number of freeze–thaw cycles, and the compressive strength loss under multi-salt soaking with freeze–thaw conditions is more serious.

Compressive strength loss decreases, then increases and then decreases with the increase in PPF dosing under multi-salt soaking and freeze–thaw conditions. After 100 freeze–thaw cycles (Figure 11), the minimum compressive strength loss (37.72%) was observed for the concrete with PPF dosing of 1.5 kg/m^3^ and the maximum compressive strength loss (72.5%) was observed for the control group. Therefore, the addition of PPF to concrete under salt freezing conditions can reduce the loss of compressive strength. Optimal dosing (1.5 kg/m^3^) exists under salt-freezing conditions.

The compressive strength loss increases, then decreases and then increases again with the increase in PPF dosing under potable and water freeze–thaw conditions. After 100 freeze–thaw cycles (Figure 12), the compressive strength of concrete with PPF dosing of 0.9 kg/m^3^ showed not only no loss but also an increase. The reason for this may be the addition of the right amount of PPF being due to the small density of fibers, and a number of reasons such as the formation of a dense “fiber network” inside the concrete. It reduces the pore connectivity in the concrete and makes the interior of the concrete denser, which alleviates the uneven distribution of internal stress in the concrete and finally effectively alleviates the loss of compressive strength of the concrete. The maximum compressive strength loss of concrete with PPF dosing was 1.5 kg/m^3^ (65.07%). Therefore, the addition of PPF to concrete under clear water and freeze–thaw conditions can reduce the loss of compressive strength, and the optimum admixture level (0.9 kg/m^3^) exists under clear water and freeze–thaw conditions.

(2) Effect of MK content

**Figure 13:**
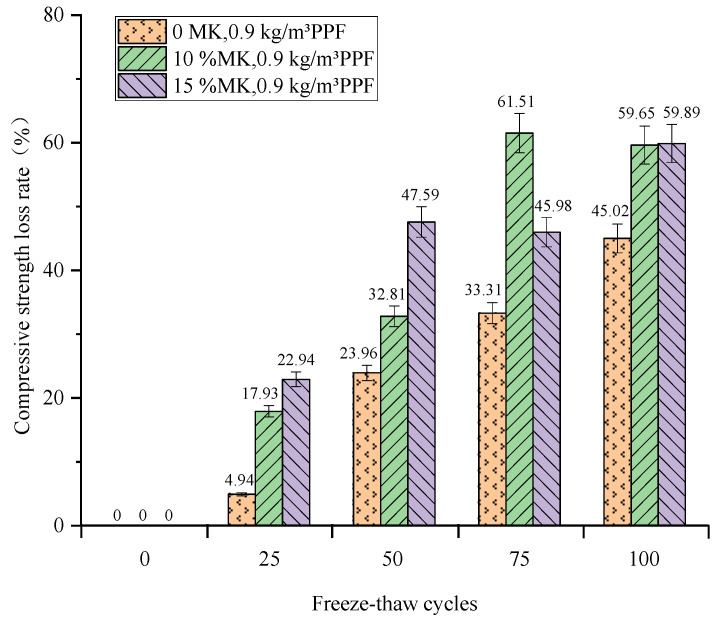
Relationship between freeze–thaw cycles and compressive strength loss rate under multi-salt soaking and freeze–thaw for MK.

**Figure 14:**
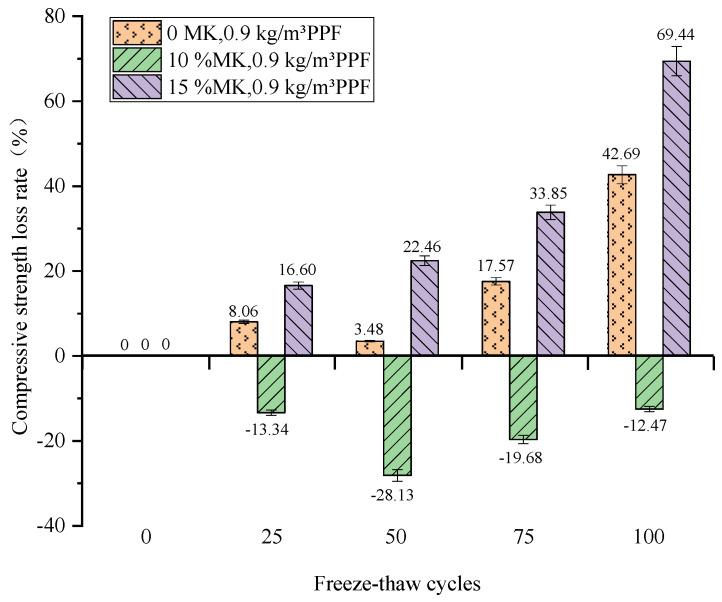
Relationship between freeze–thaw cycles and compressive strength loss rate under potable water and freeze–thaw (Note: negative values indicate increments).

The relationship between the compressive strength loss rate and the number of freeze–thaw cycles of concrete under different freeze–thaw conditions and the PPF dosage is shown in Figure 13 and Figure 14. The compressive strength of concrete under both salt and clear water and freeze–thaw conditions decreases with the increase in the number of freeze–thaw cycles from the general trend. And concrete freeze–thaw cycles make early compressive strength loss under multi-salt soaking and freeze–thaw conditions more serious.

We observed increasing compressive strength loss with increasing MK dosing under multi-salt soaking with freeze–thaw conditions. After 100 freeze–thaw cycles (Figure 13), the smallest loss of compressive strength of the control concrete was (45.02%). The compressive strength loss of concrete with 10% and 15% MK was close to 59.65% and 59.89%, respectively. Therefore, the addition of MK to concrete under multi-salt soaking and freeze–thaw conditions did not reduce the loss of compressive strength. The reason for this analysis may be that the concrete resistance to Cl^−^ penetration can be effectively improved with the increase in MK [29]. However, the chemical corrosion reactions of Mg^2+^ and SO_4_^2−^ on concrete promote each other, driving the development of its microcracks and accelerating the freeze–thaw damage effect.

The compressive strength loss decreases and then increases as the amount of MK increases under potable and water freeze–thaw conditions. After 100 freeze–thaw cycles (Figure 14), the compressive strength of the concrete with 10% MK admixture not only did not decrease but also showed an increase. Regarding an analysis of the reasons for this, the MK added to the concrete has two main effects. On the one hand, the filling effect of MK increases the compactness of concrete, optimizes the pore structure of concrete, and improves the concrete’s properties. On the other hand, the high volcanic ash property of MK promotes the hydration process of the system, which can react with the Ca(OH)_2_ produced by cement hydration and increase the content of cementitious materials in the hydration products and promote the formation of hydration products containing aluminum phases, thus filling the internal pores of concrete, which is conducive to the improvement of the concrete’s properties. 

The maximum compressive strength loss (65.07%) was observed for the concrete with 15% MK dosing. The compressive strength of concrete mixed with MK at 10% increased at all stages of the cycle. The compressive strength increased and then decreased as the number of freeze–thaw cycles increased. Therefore, the addition of MK to concrete under potable and water freeze–thaw conditions reduces the loss of compressive strength, and an optimum admixture level (10%) exists.

### 3.5. Microstructure

**Figure 15:**
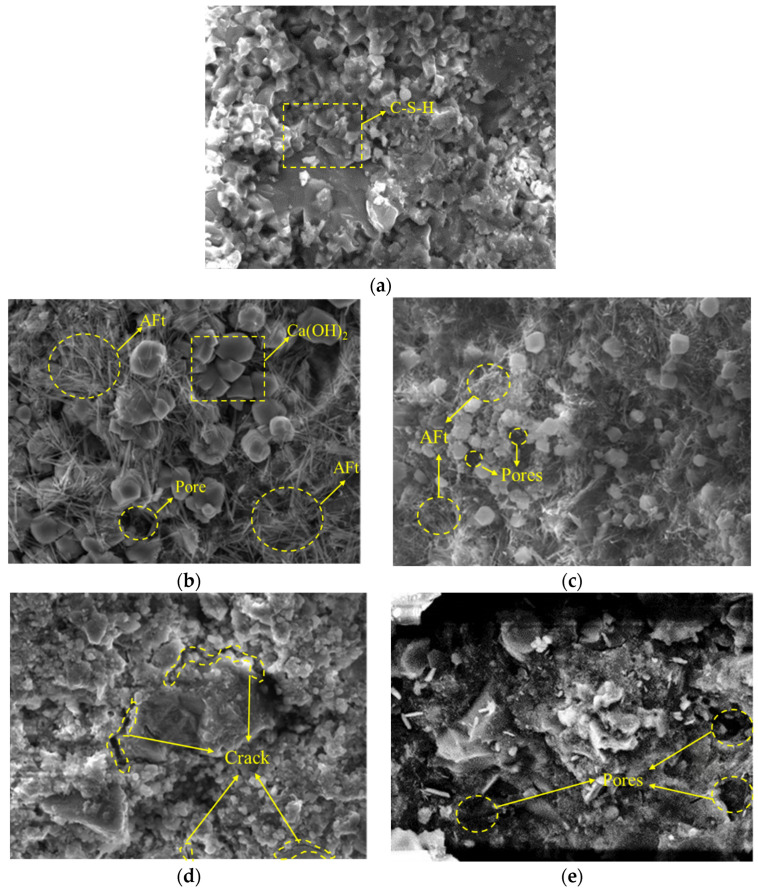
Scanning electron microscope of concrete with different freeze–thaw cycles and PPF dosages. (**a**) P-I (0 cycle); (**b**) P-I (50 cycles); (**c**) P-II (50 cycles); (**d**) P-I (100 cycles); (**e**) P-II (100 cycles).

**Figure 16:**
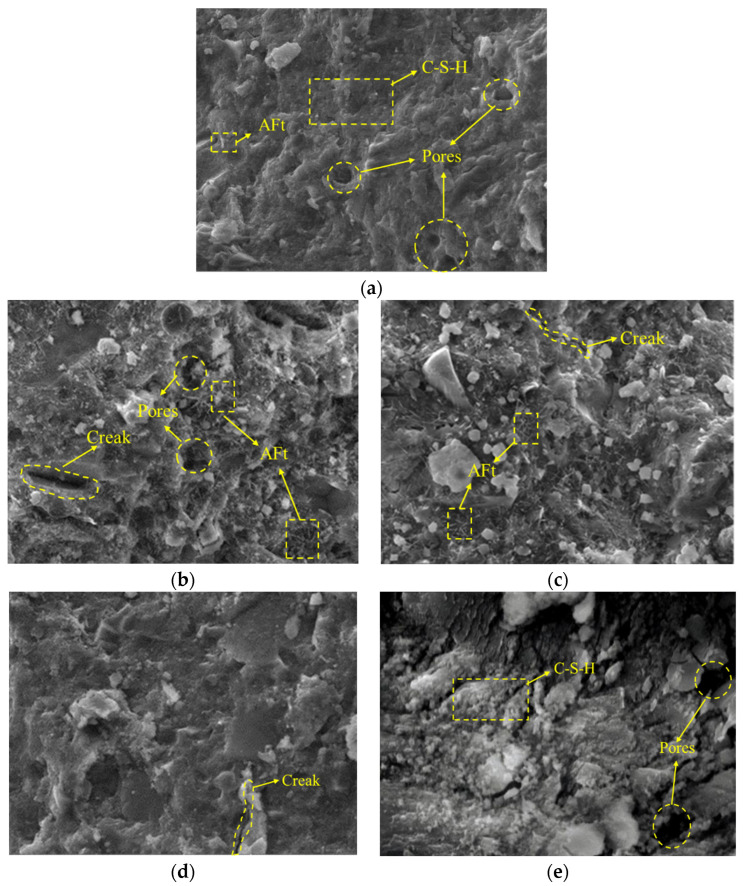
Scanning electron microscope of concrete with different freeze–thaw cycles and MK dosages. (**a**) M-I (0 cycle); (**b**) M-I (50 cycles); (**c**) M-III (50 cycles); (**d**) M-I (100 cycles); (**e**) M-III (100 cycles).

To further investigate the internal microstructure of the concrete under the compounding of PPF and MK under salt freezing, typical specimens of each group of concrete were selected for electron microscopy scanning. The microstructure diagrams are shown in Figure 15 and Figure 16.

(1) The influence of freeze–thaw cycles and PPF content under multi-salt soaking with freeze–thaw conditions.

When PPF was not incorporated, the concrete was well hydrated and structurally dense before freezing and thawing (Figure 15a). With the increase in the number of freeze–thaw and the combined effect of salt erosion, the production of calcium alumina with swelling products (Figure 15b) led to more cracks and pores inside the concrete (Figure 15d). The reason may be that the two pressures of expansion and penetration will act on the capillary pore wall at the same time. Cracks in concrete after repeated freezing and thawing penetrate each other, causing damage to the concrete structure [21].

When PPF was incorporated, compared to the concrete without PPF (Figure 15b,d) for the same number of freeze–thaw cycles. The heterogeneous distribution of PPF forms a complex three-dimensional system inside the concrete, and PPF reduces the number of primary cracks inside the concrete, thus blocking the channels for water evaporation and effectively maintaining the retention of water inside the concrete. The hydration reaction of concrete is more adequate and the internal crystallization of concrete is relatively good (Figure 15c,e). PPF can effectively assist the concrete to resist stresses and inhibit the expansion of cracks [30]. But too many fibers can undergo an agglomeration effect and reduce the frost resistance of concrete.

(2) The influence of freeze–thaw cycles and MK content under multi-salt soaking with freeze–thaw conditions.

When not mixed with MK, a large number of hydration products are in the concrete before freezing and thawing the dense structure (Figure 16a). With the increase in the number of freeze–thaw cycles and the combined effect of salt erosion, the production of acicular calcium vanadinite (Figure 16b) leads to an increase in volume, resulting in stress. Cracking occurs when the stress exceeds the tensile stress in the concrete.

When MK was added, compared with the concrete without MK (Figure 16b,d), under the same number of freeze–thaw cycles, hydrogen ions or other aggressive ions from the external environment enter the concrete interior through the pores and first neutralize with the free OH^−^ ions. Therefore, the hydration reaction of concrete is weakened after MK replaces the cement, and the content of Ca(OH)_2_ generated by hydration is reduced. The decrease in free OH- in the pores of concrete leads to a decrease in the PH value, which destroys the stable environment for the existence of hydration products. Some of the hydration products’ structural damage occurred during decomposition [31], concrete coarse cracks increased, and the structural damage was also more severe (Figure 16c,e). This further confirms that the destruction of the alkaline environment leads to the reduction of hydration products, resulting in the weakened resistance of concrete to salt freezing.

## 4. Conclusions

The effects of MK and PPF dosing on the physical and mechanical properties and microstructure of concrete under multi-salt soaking with freeze–thaw and potable water freezing and thawing conditions were investigated. The following conclusions were obtained:

1. Compared to potable water freezing and thawing, salt freezing is more corrosive to concrete materials. The compressive strength of concrete with 0.9 kg/m^3^ PPF after 100 freeze–thaw cycles increased by 12.47% under potable water and freeze–thaw conditions, and its loss of compressive strength was 59.65% under salt freezing conditions.
2. The changes in appearance, quality, relative dynamic modulus of elasticity and compressive strength under multi-salt soaking with freeze–thaw conditions are a good reflection of the damage to the concrete. A combination of the four can be used as a criterion to determine concrete damage.
3. PPF can effectively enhance the erosion resistance of concrete under multi-salt soaking with freeze–thaw conditions. MK can only improve the erosion resistance of concrete under potable water conditions. The relative dynamic modulus of elasticity of concrete with 10% MK was 54.46% at 100 cycles of freezing and thawing in potable water, which was much higher than that of the control group (22.32%) without MK.
4. Concrete with a small amount of PPF added under salt freezing conditions had fewer cracks after a comparative analysis of the SEM images. Fibers can effectively assist the concrete to resist stress, inhibit the expansion of cracks, and improve the concrete’s resistance to freezing and thawing. The addition of MK destroys the alkaline environment inside the concrete and reduces the generation of hydration products resulting in the weakened ability of concrete to resist multi-salt soaking with freeze–thaw tests.
5. Improvement to the erosion resistance of concrete by multiple admixtures including PPF and MK can be considered in future salt freezing studies.

## Figures and Tables

**Table 1:** Chemical composition of metakaolin and cement.

Material	Composition w/%				Fineness/			
	SiO_2_	Al_2_O_3_	Fe_2_O_3_	CaO + MgO	K_2_O + Na_2_O	Other	Ignition Loss	M^2^·kg^−1^
MK	51.18	41.59	0.47	3.4	3.32	0.04	0.71	4665
Cement	20.43	5.38	3.95	65.15	1.01	4.08	1.38	350

**Table 2:** Preparation of multi-salt soaking.

Erosion Solution	Salt Type and Dosage/(g·L^−1^)				Multi-Salt Soaking Concentration/%
	Na_2_SO_4_	MgCl_2_	NaHCO_3_	NaCl	
Benchmark	0.97	3.74	0.82	29.61	3.51

**Table 3:** Mix proportion of concrete.

Specimen Number	W/B	Mix Proportion/(kg·m^−3^)								Slump/mm
		Cement	Water	Sand	Gravel	Water Reducing Admixture	Air Entraining Agent	MK	PPF	
P-I	0.45	446.4	223	645	1145	5.95	0.036	49.6	-	70~90
P-II	0.45	446.4	223	645	1145	6.45	0.036	49.6	0.6	
P-III	0.45	446.4	223	645	1145	5.95	0.036	49.6	0.9	
P-IV	0.45	446.4	223	645	1145	3.47	0.036	49.6	1.5	
M-I	0.45	496	223	645	1145	2.98	0.036	-	0.9	
M-II	0.45	446.4	223	645	1145	5.95	0.036	49.6	0.9	
M-III	0.45	421.6	223	645	1145	3.97	0.036	74.4	0.9	

**Table 4:** The corrosion of concrete under multi-salt soaking and freeze–thaw with different dosages of PPF.

Freeze–Thaw Cycles	Specimen Number			
	P-I	P-II	P-III	P-IV
0	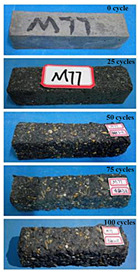	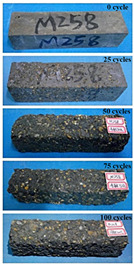	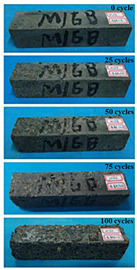	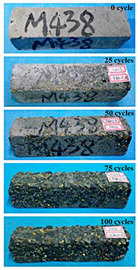
25				
50				
75				
100				

**Table 5:** The corrosion of concrete under potable water and freeze–thaw with different dosages of PPF.

Freeze–Thaw Cycles	Specimen Number			
	P-I	P-II	P-III	P-IV
0	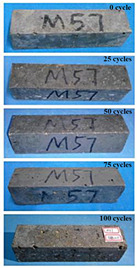	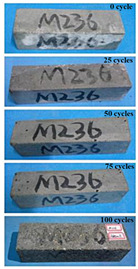	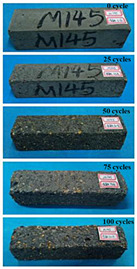	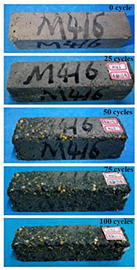
25				
50				
75				
100				

**Table 6:** The corrosion of concrete under multi-salt soaking and freeze–thaw with different dosages of MK.

Freeze–Thaw Cycles	Specimen Number		
	M-I	M-II	M-III
0	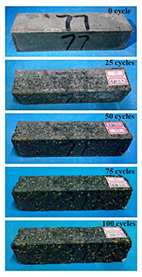	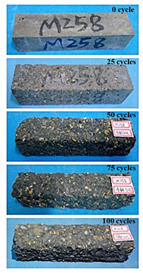	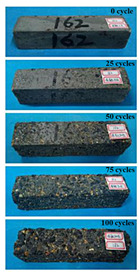
25			
50			
75			
100			

**Table 7:** The corrosion of concrete under potable water and freeze–thaw with different dosages of MK.

Freeze–Thaw Cycles	Specimen Number			
	M-I	M-II		M-III
0	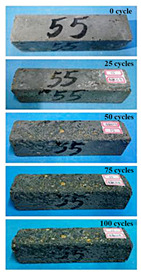	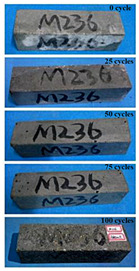	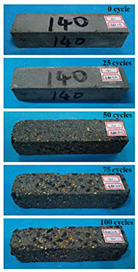	
25				
50				
75				
100				

## Data Availability

Data sharing not applicable. No new data were created or analyzed in this study. Data sharing is not applicable to this article.
